# Nearly half of HIV-positive children attending public health facilities are suffering from chronic under-nutrition in conflict-affected zones of Southern Ethiopia

**DOI:** 10.3389/fnut.2024.1356931

**Published:** 2024-04-17

**Authors:** Awoke Abraham, Joseph Kawuki, Tesfaye Aragie, Cherinet Elias, Zewudu Birahanu, Desalegn Dawit, Eskinder Israel

**Affiliations:** ^1^Division of Nutrition, Maternal and Child Health Unit, Wolaita Zone Health Department, Sodo, Ethiopia; ^2^Centre for Health Behaviours Research, Jockey Club School of Public Health and Primary Care, The Chinese University of Hong Kong, Shatin, Hong Kong SAR, China; ^3^Department of Public Health, Private Sector Support Officer, Marie Stopes International Ethiopia Reproductive Choices, Hawassa, Ethiopia; ^4^School of Public Health, College of Health Science and Medicine, Wolaita Sodo University, Sodo, Ethiopia

**Keywords:** chronic malnutrition, antiretroviral, pediatrics, HIV/AIDS, conflict, Southern Ethiopia

## Abstract

**Background:**

In combination with HIV infection, malnutrition is a complicated medical condition with high morbidity and mortality rates in affected children due to a variety of socioeconomic and medical etiological variables. To combat this, information from a range of contexts is required, but there is little evidence, particularly about the nutritional status of under 15 living with HIV in impoverished communities such as conflict affected areas. Therefore, in this study the magnitude and related factors of stunting among under 15 children antiretroviral therapy at public health facilities was assessed.

**Methods:**

An institution-based cross-sectional study was conducted among under 15 children living with HIV in conflict-affected zones of Southern Ethiopia. After providing written informed consent to study participants, data were collected using an interviewer-administered questionnaire and anthropometric measurements. Bivariable and multivariable logistic regression models were used to identify factors associated with nutritional status, using SPSS Version 25.

**Results:**

Of the 401 participants, 197 (49.1%, 95% CI: 0.44, 0.54) had height-for-age *z*-score ≤ -2. In the multivariable analysis, larger household size (AOR = 1.58, 95% CI: 1.04–2.40), dietary diversity (AOR = 1.78; 95% CI: 1.07–2.96) and having a history of recurrent diarrhea (AOR = 1.96; 95% CI: 1.07–3.59) were significantly associated with chronic under nutrition.

**Conclusions:**

The prevalence found in this study was high when compared with the stunting target set in SDG, which states to end all forms of malnutrition In order to mitigate the negative health effects of diarrhea during HIV therapy, extra attention needs to be paid to facilitate timely detection and on-going monitoring. Nutrition programs in conflict-affected areas need to consider households with larger family sizes and/or routinely having fewer food groups.

## Background

Stunting is low height for age which is an indicator of chronic under-nutrition (1). Globally, chronic undernutrition affects both adults and children and is a worldwide public health concern (2). Thus, 22% of children under 5 years of age in 2019 and 23% of school-age children (5–14 years) in 2020 were stunted (3, 4). Similarly, the prevalence of stunting in the Sub-Saharan Africa (SSA) region was 32.3% (5). In Ethiopia, stunting also remains as high as 49.7% which calls for continued adequate investment in nutrition (6). Stunting significantly increased the risk of childhood morbidity and mortality (7, 8). Further, stunted children also have a higher risk of being overweight or obese later in life, putting them at risk of chronic disease in adulthood (7, 9). More than 35% of child mortality (10), 11% of the world's disease burden (11), and abnormalities including cognitive decline, chronic diseases, and growth failure are all caused by under nutrition (10).

Acquired immune deficiency syndrome (AIDS), is a disease caused by a retrovirus, the human immunodeficiency virus (HIV), which attacks and impairs the body's natural defense system against disease and infections (12). About two-thirds of the 38.4 million people living with HIV (PLWH), 45% of the 1.5 million new cases, and 43% of the 650,000 AIDS-related mortality recorded worldwide in 2021 were from SSA (13). When HIV/AIDS and malnutrition coexist in the same individual, they can be fatal combos that feed off one another. Malnutrition increases susceptibility to infection by causing immune dysfunction in manifold ways. Weakened immune function can increase HIV replication and hasten the development of HIV illness into AIDS (14). When people with HIV/AIDS are not receiving treatment, they are more likely to become malnourished (15), which raises their risk of dying when antiretroviral medication (ART) is started (14). Furthermore, people with untreated or advanced HIV/AIDS often have weakened immune systems, which leaves them vulnerable to a range of opportunistic infections (16).

Despite Ethiopia being the oldest sovereign state on the African continent, conflict has become the norm (17). The political unrest and instability that invariably accompanies leadership changes instill a sense of uneasiness in citizens. It wouldn't be an overstatement to suggest that war and conflict have characterized Ethiopia's history (18). Renewing hostilities in most regions, including Southern Ethiopia, between non-state armed groups and regional security forces over resource competitiveness and land disputes have resulted in violent confrontations that have forced people out of their homes in recent years (19). Conflict halts farming and destroys people's livelihoods, thereby diminishing households' and communities' capacity to cope with the basic root causes of chronic malnutrition (20, 21). Interruptedly conflict may block food aid from reaching populations at risk of malnutrition (22, 23).

Maintaining optimal nutrition is still a significant difficulty for children living with HIV; factors that contribute to this problem include insufficient dietary intake, side effects of antiretroviral therapy (ART), and HIV infection itself (24). Although there have been some studies assessing the nutritional status of children living with HIV in Ethiopia, they have focused on either the general nutritional status or acute malnutrition but none specifically exploring chronic malnutrition in conflict-affected settings (25–29). Although chronic malnutrition remains a major public health problem and a major cause of morbidity and mortality in HIV-positive children, there is a deficit of studies assessing the nutritional status and associated factors in HIV-positive children in conflict-affected zones of Southern Ethiopia.

The current study, therefore, aimed to assess determinants of chronic malnutrition among children receiving antiretroviral therapy (ART) in health facilities of conflict-affected zones of Southern Ethiopia. The findings from this study may be helpful for public health authorities and other stakeholders to rethink the current health and nutritional strategies and programs for this vulnerable population group. The study findings may also be important for parents, caretakers and children in familiarizing the factors associated with stunting, and ways of redress.

## Methodology

### Study area, period, and design

From 9th June to 29th July 2022 a health facility-based cross-sectional study was conducted in Amaro, Burji, Derashe, Ale, and Konso zones, in Southern Ethiopia. These areas were respectively 357, 546, 550, and 522 km from south of Addis Ababa, the capital of Ethiopia.

Based on the latest population projection of the Central Statistics Agency of Ethiopia, the population of the study area was projected to be 3,157,673 (with 473 597 urban (15%) and 2,684,076 rural (85%) residents) (30). This area is one of inter-communal conflict-affected Ethiopian zones (31). In this study area, there are 21 health facilities delivering ART services during the study period.

### Study population

All under 15, HIV-positive children receiving ART in health facilities of conflict-affected zones of South Ethiopia regional State were the study population.

### Inclusion and exclusion criteria

All HIV-infected children who had ART follow-up in randomly selected health facilities of conflict-affected zones were included. Children with incomplete baseline medical information had physical malformation (kyphosis and lordosis) and/or were seriously ill were excluded from the study.

### Sample size determination

Our target sample size was 419. This sample size was determined using a formula for single population proportion by considering a 45.2% proportion (p) of chronic malnutrition; 5% margin of error; 10% non-response rate; and 95% confidence intervals (CI) (32). The same approach was used by similar studies (32, 33). The study was conducted at eight selected healthcare facilities. Initially, a sampling frame was prepared using the patient's medical registration number from each health facility's ART registration log. Following that, the total sample size was distributed proportionally to each facility. The study participants were then picked from each of the identified healthcare facilities using a computer-generated basic random sampling procedure.

### Variables

#### Outcome variable

The outcome variable was stunting among HIV- positive children on ART in conflict-affected zones of Southern Ethiopia. Stunting was defined as a child having either a height for age *Z*-score below −2 standard deviation (SD) of the median value of WHO standard (33). Data were collected by using standard procedures.

#### Independent variables

These included socio-demographic and clinical-related variables. Socio-demographic variables such as age, sex and residence of the child, age of mother, educational status of household head, marital status of household head, occupation of household head, household family size, dietary diversity, main source of family food, and counseling on child nutrition, were considered. In addition, several *clinical-related variables* were considered; Immunodeficiency status (CD4 count), viral load, WHO clinical stage, exposure to prophylaxis, opportunistic infections, recurrent diarrhea, oropharengeal disease, adherence to ART, duration on ART, anemia history, and history of tuberculosis.

### Operational definitions

*HIV-Positive Children:* Children aged < 15 years with confirmed HIV infection (27).

Dietary diversity: The number of reported different foods and food groups consumed in an individual over 24 h. This includes food groups consumed outside the home and < 4 and greater or equal to 4 food groups are fair/poor and good dietary diversity, respectively (34).

*History of Opportunistic Infections*: child with HIV-related infections other than chronic diarrhea and disease; within the last 6 months before stunting and wasting diagnosis (27).

*History of Oropharengeal Disease*: A child with oral ulcer or candidiasis/oral thrush; within the last 6 months before stunting and wasting diagnosis (27).

*History of recurrent Diarrheal Disease:* A child with diarrheal disease of 30 days or more duration; within the last 6 months before diagnosis (27).

*Adherence to ART: This was categorized as Good, Fair and Poor treatment adherence if a child* missed ≤ 2, < 2 ≤ 5, and >5 medical prescriptions, respectively (35).

### Data collection procedures

A total of eight research assistants and three supervisors assisted with data collection. They were at least diploma holder health professionals who spoke the local language and had previous experience in data collection. Training about the objectives of the study, the contents of the tool and data collection procedures were given to data collectors and supervisors for 4 days at their respective zonal centers.

Height was measured by using a stadiometer. Sociodemographic data from mother/caregiver of child were collected by face-to-face interviews and clinical characteristics were taken from medical records. At the time of actual data collection, nutritional advice was given to all caregivers. The assigned supervisors and principal investigator closely monitored and supervised the whole data collection process.

### Statistical analysis

Data analysis was done using Statistical Package for Social Sciences (SPSS) Version 25. The anthropometric measurements were converted into *Z*-scores using WHO Anthro and WHO Anthro++ software version 3.2.2. Frequencies and cross-tabulation were used to check for missing values of variables and to describe the study population concerning relevant variables. Moreover, percentages, proportions, and summary statistics were used in summarizing the study population characteristics. Binary logistic regression analysis was conducted to assess the independent association of factors against the outcome variable. Variables with *p*-values < 0.25 in the bi-variable analysis were entered into the Multivariable Logistic Regression model to control for the effect of confounders and identify significant factors. The adequacy of the model to fit the outcome variable with the predictors was checked using the Hosmer-Lemeshow test for goodness of fit, and the value was 0.39, which was in the good range. In the multivariable analysis, variables with *p*-values < 0.05 were considered statistically significant, and we reported their respective adjusted odds ratios (AOR) with correspondence 95% confidence intervals (CI).

### Patients and the public involvement

Patients and the public were not involved in the design of the study, the conduct of the study or the dissemination of the findings.

## Results

### Socio-demographic characteristics of study participants

Out of the 419 potential participants approached, 401 agreed and successfully participated in this study with a response rate of 95.7%. Of the total (401) respondents nearly half (50.6%) were rural dwellers, 220 (54.9%) were married, and 209 (52.1%) respondents had five or less family size. Regarding the index child characteristics 212 (52.9%) children were male, and 156 (38.9%) children were ≤ 60 months of age while 129 (32.2%) were 60–120 months of age (Table 1). In addition, 297 (74.1%) households had poor dietary diversity, with the majority (46.1%) of households obtaining their food from aid, 125 (31.17%) buying from the market, and 91 (22.7%) from farming. However, about 151 (37.7%) participants responded that they did not get nutritional counseling.

**Table 1:** Socio-demographic characteristics of HIV positive under 15 years of age attending ART clinics of health facilities of conflict-affected areas in South Ethiopia, 2022.

**Variable**	**Category**	**Frequency, *n* (%), *N* = 401**
Age of child in months	≤ 60	156 (38.9)
	>60 to ≤ 120	129 (32.2)
	120 to 179	116 (28.9)
Sex of child	Male	212 (52.9)
	Female	189 (47.1)
Sex of household head	Male	248 (61.8)
	Female	153 (38.2)
Age of mother	≤ 20	146 (63.6)
	>20	255 (36.4)
Educational level of household head	No education	98 (24.4)
	Primary	126 (31.4)
	Secondary	95 (23.7)
	Certificate and above	82 (20.5)
Occupation of household head	Not employed	102 (25.4)
	Employed with salary	133 (33.2)
	Casual labor	84 (20.9)
	Small scale trading	82 (20.4)
Residence	Urban	163 (40.6)
	Semi-urban	40 (10.0)
	Rural	198 (49.4)
Marital status	Married	220 (54.9)
	Divorced/separated/widowed	110 (27.4)
	Single	71 (17.7)
Family size	1–4	192 (47.9)
	≥5	209 (52.1)
Source of food for household	Farming	91 (22.7)
	Buying	125 (31.2)
	Aid	185 (46.1)
Exposure to nutritional counseling	No	151 (37.7)
	Yes	250 (62.3)
Dietary diversity	Good	104 (25.9)
	Poor	297 (74.1)

### Clinical related characteristics

Majority of children, 239 (59.6%) were not given prophylaxis and 194 (48.4%) of them had stayed < 60 months on ART. Two hundred and forty-seven of children were in WHO clinical stage I and II category, and the rest 152 (37.9%) of them were in clinical stage III and IV. More than half, 219 (54.6%) of children had experienced opportunistic infection (such as oropharengeal disease in 60.3%, chronic diarrhea in 85.8%, and tuberculosis in 38.2% of children; Table 2).

**Table 2:** Clinical characteristics of HIV positive under 15 years of age children attending ART clinics of conflict affected zones in South Ethiopia, 2022.

**Variable**	**Category**	***N* (%)**
Exposure to prophylaxis	No prophylaxis given	239 (59.6)
	Prophylaxis given	162 (40.4)
Duration on ART in month	≤ 60	194 (48.4)
	>60	207 (51.6)
Adherence to ART	Poor	302 (75.3)
	Good	55 (13.7)
	Fair	44 (11.0)
Viral load (the number of HIV copies in a milliliter of blood)	0–999	291 (72.6)
	≥1,000	110 (27.4)
CD4 count (in cells/mm^3^)^a^	< 350	146 (61.5)
	≥350	154 (38.5)
Who clinical stage^b^	Stage I ∧ Stage II	247 (61.6)
	Stage III ∧ Stage IV	152 (37.9)
History of anemia	No	186 (46.4)
	Yes	215 (53.6)
History of opportunistic infections	No	182 (45.4)
	Yes	219 (54.6)
History of oropharengeal disease	No	159 (39.7)
	Yes	242 (60.3)
History of chronic diarrhea	No	57 (14.2)
	Yes	344 (85.8)
History of tuberculosis	No	248 (61.8)
	Yes	153 (38.2)

^a^1 missing value.

^b^2 missing values.

ART, Antiretroviral therapy; HIV, Human immunodeficiency virus; WHO, World health organization; CD4, Cluster of differentiation 4.

### Prevalence of stunting

**Figure 1:**
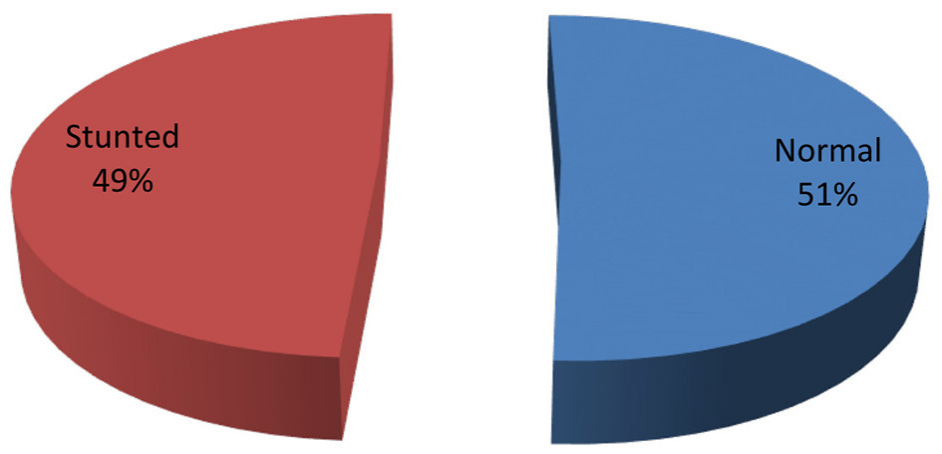
Prevalence of stunting among HIV positive under-15 years children attending ART clinics in health facilities of conflict affected areas in South Ethiopia, 2022.

Of the 401 HIV positive children in this study, 197 (49.1%, 95% CI = 0.44–0.54) were found to be stunted i.e., HAZ < −2 and 55 (13.7%) were severely stunted; HAZ < −3 (Figure 1). The mean height of the study population was 109.5 cm with standard deviation (SD) = ±18.8.

### Factors associated with stunting

This study examined a relationship between different participant characteristics and stunting. In the bivariable analysis, occupation of household head, dietary diversity, household size, duration on ART, history of aneamia, exposure to prophylaxis, viral load, WHO clinical stage and history of chronic diarrhea showed independent significant association with stunting among HIV positive children. After adjusting for possible confounders in the multivariable analysis, household size (AOR = 1.58; *P*-value = 0.02), dietary diversity (AOR = 1.78; *P*-value = 0.03), and history of chronic diarrhea (AOR = 1.96; *P*-value = 0.03) had statistically significant association with stunting of HIV positive children (Table 3).

**Table 3:** The bivariable and multivariable logistic regression analysis of associated factors of stunting at conflict affected zones of Southern Ethiopia, 2022.

**Variable**	**Crude odds ratio, COR (95% CI)**	***p*-value**	**Adjusted odds ratio, AOR (95% CI)**	***p*-value**
Age of child in months		0.77	N/A	
120 to 179	1			
6 to ≤ 60	1.18 (0.73–1.91)			
>60 to ≤ 120	1.17 (0.71–1.93)			
Sex of child		0.39	N/A	
Female	1			
Male	1.19 (0.80–1.76)			
Sex of household head		0.91	N/A	
Female	1			
Male	1.02 (0.68–1.53)			
Age of mother		0.40	N/A	
>20	1			
≤ 20	1.19 (0.79–1.79)			
Educational level of household head		0.68	N/A	
Certificate and above	1			
No education	1.43 (0.80–2.58)			
Primary	1.14 (0.65–1.99)			
Secondary	1.19 (0.66–2.15)			
Occupation of household head		0.12		0.32
Small scale trading	1			
Not employed	0.83 (0.48–1.44)		1.15 (0.62–2.11)	
Employed with salary	1.62 (0.88–3.00)		0.78 (0.44–1.40)	
Casual labor	1.15 (0.64–2.05)		1.32 (0.68–2.53)	
Residence		0.96	N/A	
Urban	1			
Semi-urban	0.94 (0.47–1.88)			
Rural	1.01 (0.51–1.97)			
Marital status		0.59	NA	
Married	1			
Divorced/separated/widowed	1.31 (0.72–2.39)			
Single	1 (0.63–1.58)			
Family size		< 0.01		**0.02**
≥5	1			
1–4	1.70 (1.15–2.52)		**1.58** (1.04–2.40)	
Source of food for household		0.38		
Aid	1			
Farming	1.08 (0.65–1.78)			
Buying	1.37 (0.87–2.17)			
Dietary diversity		< 0.01		**0.03**
Good	1			
Poor	2.30 (1.45–3.64)		**1.78** (1.07–2.96)	
Nutrition counseling		0.50	NA	
Yes	1			
No	1.16 (0.77–1.73)			
Exposure to prophylaxis		0.16		0.63
Prophylaxis given	1			
No prophylaxis given	1.34 (0.90–2.00)		1.11 (0.71–1.72)	
Duration on ART in month		< 0.01		0.06
≤ 60	1			
>60	1.85 (1.24–2.74)		1.46 (0.95–2.24)	
Adherence to ART		0.66		
Good	1			
Poor	1.01 (0.54–1.91)			
Fair	0.77 (0.35–1.72)			
Viral load (the number of HIV copies in a milliliter of blood)		0.24	0.93 (0.56–1.54)	0.78
≥1,000	1			
0–999	1.31 (0.84–2.03)			
CD4 count (in cells/mm^3^)		0.39		0.06
≥350	1			
< 350	0.84 (0.56–1.26)		0.66 (0.43–1.02)	
WHO clinical stage		0.07		0.07
Stage I ∧ Stage II	1			
Stage III ∧ Stage IV	1.45 (0.96–2.17)		1.49 (0.98–2.27)	
History of anemia		< 0.01		0.08
No	1		1	
Yes	1.70 (1.14–2.53)		1.46 (0.95–2.24)	
History of opportunistic infections		0.86	N/A	
No	1			
Yes	0.97 (0.65–1.43)			
History of oropharengeal disease		0.92	NA	
No	1			
Yes	1.02 (0.68–1.52)			
History of recurrent diarrhea		0.03		**0.03**
No	1			
Yes	1.93 (1.08–3.43)		**1.96** (1.07–3.59)	
History of tuberculosis		0.60	NA	
No	1			
Yes	1.11 (0.74–1.67)			

The bolded values indicate significant association in multivariate logistic regression.

Based on the multivariable analysis children from households with family size of five or more members were 1.58 times more likely to be stunted than children from family size of < 5 members (AOR = 1.58; 95% CI: 1.04–2.40). Similarly, children who had poor dietary diversity practice were 1.78 times more likely to be stunted than children who had good dietary diversity practice (AOR = 1.78; 95% CI: 1.07–2.96). Moreover, children who had a history of chronic diarrhea were 1.96 times more likely to be stunted than those with no history of chronic diarrhea (AOR = 1.96; 95% CI: 1.07–3.59; Table 3).

## Discussion

The prevalence and associated factors of stunting among HIV-infected children in conflict affected zones of Southern Ethiopia were investigated in this study. Our study showed that nearly half (49.1%) of HIV-positive children were stunted. This prevalence was higher than that reported from other studies in Adama, Amhara region, and a previous study in Southern Ethiopia (32, 33, 36). Researches indicated that children were disproportionately affected in environments where there is conflict (37, 38). Most chronically malnourished people on the planet and three out of every four stunted children live in conflict-affected countries (39). When compared to the sustainable developmental goal (SDG) stunting target, the current study's findings indicate that study participants were in worst health status. Ending all forms of malnutrition, including achieving by 2025 the internationally agreed targets on stunting in children under 5 years of age, and addressing the nutritional needs of adolescents was stated (40). Reversing the high prevalence of under-nutrition that has been documented requires stakeholders' joint effort. The implementation of dietary counseling, regular nutritional assessments, and financial and in-kind nutritional support should be reinforced, observed, and assessed.

This study showed that the larger the family size, the higher the likely hood of child stunting. Previous research findings corroborate this conclusion (41–44). As the number of family members in a household increases, the amount of childcare and food consumption will decline, particularly in families where there is not enough food to go around, which exacerbates the nutritional impact and HIV infection process (45–47). The food security of HIV affected households is impaired by a number of factors including the synergy generated by hosting a HIV positive family member (48).

This study has also showed an association of dietary diversity with chronic malnutrition in which children with poor dietary diversity are more likely to be stunted than their counter parts that get good dietary diversity. Consistent finding was reported from previous studies conducted in Hawasa, as well as Amhara regional state public hospitals, Ethiopia (32, 49). The reason could be that an HIV patient who did not receive a variety of foods that are meant to strengthen and/or improve immunity will be immune-compromised due to the infection itself and will be exposed to various other infections, which ultimately lead to the development of a chronic form of stunted malnutrition (50). Furthermore, the poor dietary diversity could be derived by conflict and unrest that disrupt food chain (51), affect people's ability to produce, exchange and access food (52). However, it has been recommended that as HIV-positive individuals have additional energy requirements, particularly children who have more demand to ensure rapid physical growth and development, diversifying their diet is by far an essential part for the care of people living with HIV (53).

Our study revealed that children who had history of chronic diarrhea were about two times likely to be stunted than their counterparts. This finding was in line with reports from different settings of both Ethiopia and overseas (14, 54–56). Diarrhea results in malabsorption of basic macronutrients including carbohydrate (57, 58) and protein (59). Older studies reported that in protein malnutrition, the absorption of dietary vitamin A from the intestinal tract was impaired (60–62). The risk of deficiency of crucial micronutrients for child growth and development like zinc (63), vitamin A (64–66) was enhanced by diarrhea. In addition, conflicts harm medical systems and public health in ways that last much beyond the actual fighting phase, which makes it easier for illnesses like diarrhea to spread (67–69). Therefore, chronic diarrheal history was an indication of previous poor nutrient intake worsened by effects of conflict.

### Study strengths and limitations

This research allowed accessing a number of variables at the same time by using multicenter study and adequate sample size. Addressing relatively remote health facilities can also be taken as strength. However, during the interpretation of the findings of this study one has to consider the following limitations. Due to the cross-sectional design of the study, it could not establish the possible cause and effect relationship between independent and dependent variables In addition, since data collection was conducted during a rainy season which is usually associated with a surge in common childhood water-borne illness, it was difficult to discover the seasonal variations. Therefore, we recommend future researchers to consider seasonal patterns.

## Conclusions

The current study finding revealed a higher prevalence of chronic malnutrition among HIV-positive children in conflict affected zones of Southern Ethiopia. As compared to the stunting target set in sustainable developmental goal (SDG), which states to end all forms of malnutrition, our finding was in worst status. In order to mitigate the negative health effects of diarrhea in children during HIV therapy, extra attention needs to be paid to facilitate timely detection and on-going monitoring. Nutrition programs in conflict affected areas need to consider households with larger family size and/or routinely having a fewer food groups.

## Data availability statement

The raw data supporting the conclusions of this article will be made available by the authors, without undue reservation.

## Ethics statement

Ethical clearance was initially obtained from the Institutional Review Board (IRB) of Wolaita Sodo University, College of Medicine and Health Science with Ref. No: CRCSD105/02/14. The patients/participants [legal guardian/next of kin] provided written informed consent to participate in this study.

## Author contributions

AA: Conceptualization, Formal analysis, Funding acquisition, Investigation, Methodology, Project administration, Software, Supervision, Writing—original draft, Writing—review & editing. JK: Investigation, Methodology, Supervision, Validation, Visualization, Writing—original draft, Writing—review & editing. TA: Data curation, Resources, Software, Writing—original draft, Writing—review & editing. CE: Funding acquisition, Software, Writing—original draft, Writing—review & editing. ZB: Data curation, Funding acquisition, Investigation, Project administration, Writing—original draft, Writing—review & editing. DD: Formal analysis, Funding acquisition, Software, Writing—original draft, Writing—review & editing. EI: Funding acquisition, Investigation, Project administration, Writing—original draft, Writing—review & editing.

## References

[1] de Onis M Onyango AW. WHO child growth standards. Lancet. (2008) 371:204. 10.1016/S0140-6736(08)60131-2 18207015

[2] Blössner M Onis MD Prüss-üstün A Campbell-lendrum D Corvalán C Woodward A. Malnutrition Quantifying the Health Impact at National and Local Levels. Geneva: WHO (2005).

[3] UNICEF WHO The The World Bank. Levels and Trends in Child Malnutrition: Key Findings of the 2019 Edition of the Joint Child Malnutrition Estimates. Geneva: World Health Organization (2019). p. 1–15.

[4] WHO. UNICEF/WHO/World Bank Group - Joint Child Malnutrition Estimates: Levels and Trends in Child Malnutrition: Key Findings of the 2020 Edition. Geneva: World Health Organization (2020). p. 1–15. Available online at: https://www.who.int/publications-detail/jme-2020-edition (accessed December 20, 2022).

[5] WHO. Conflict, COVID-19, Climate Change and Growing Inequalities Are Converging to Undermine Food Security Worldwide. Geneva: World Health Organization (2022) 1:10017.

[6] Abate BB Aragie TG Tesfaw G. Magnitude of underweight, wasting and stunting among HIV positive children in East Africa: a systematic review and meta-analysis. PLoS ONE. (2020) 15:e0238403. 10.1371/journal.pone.0238403 32941443 PMC7498078

[7] Black RE Victora CG Walker SP Bhutta ZA Christian P De Onis M. Maternal and child undernutrition and overweight in low-income and middle-income countries. Lancet. (2013) 382:427–51. 10.1016/S0140-6736(13)60937-X 23746772

[8] Winterfeld A. Improving Child Nutrition. Vol. 18. NCSL legisbrief (2010). p. 1–2. 20196250

[9] Soliman A De Sanctis V Alaaraj N Ahmed S Alyafei F Hamed N. Early and long-term consequences of nutritional stunting: from childhood to adulthood. Acta Biomed. (2021) 92:1–12. 10.23750/abm.v92i1.11346 33682846 PMC7975963

[10] Black MM Walker SP Wachs TD Ulkuer N Gardner JM Grantham-McGregor S. Policies to reduce undernutrition include child development. Lancet. (2008) 371:4545. 10.1016/S0140-6736(08)60215-9 18262026

[11] Grantham-McGregor S Cheung YB Cueto S Glewwe P Richter L Strupp B. Developmental potential in the first 5 years for children in developing countries. Lancet. (2007) 369:60–70. 10.1016/S0140-6736(07)60032-4 17208643 PMC2270351

[12] Chen YD. Acquired immune deficiency syndrome. Zhonghua Liu Xing Bing Xue Za Zhi. (1984) 5:367–9. 6532535

[13] Moyo E Moyo P Murewanhema G Mhango M Chitungo I Dzinamarira T. Key populations and Sub-Saharan Africa's HIV response. Front Public Heal. (2023) 11:1079990. 10.3389/fpubh.2023.1079990 37261232 PMC10229049

[14] Duggal S Chugh T Das Duggal AK. HIV and malnutrition: effects on immune system. Clin Dev Immunol. (2012) 2012:784740. 10.1155/2012/784740 22242039 PMC3254022

[15] K. Sashindran V, Thakur R. Malnutrition in HIV/AIDS: aetiopathogenesis. Nutr HIV/AIDS Implic Treat Prev Cure. (2020) 2020:90477. 10.5772/intechopen.90477

[16] Sashindran VK Thakur R. Malnutrition in HIV/AIDS : Aetiopathogenesis. InTech (2020). p. 1–24.

[17] Bélair J. Ethnic federalism and conflicts in Ethiopia. Can J Afr Stud. (2016) 50:295–301. 10.1080/00083968.2015.1124580

[18] Geda A. Does Conflict Explains Ethiopia's Backwardness? Yes! and Significantly Seattle, WA: Allen Institute. (2004).

[19] Norwegian Refugee Council. Internal Displacement Monitoring Center. Geneva (2022).

[20] Raleigh C Choi HJ Kniveton D. The devil is in the details: an investigation of the relationships between conflict, food price and climate across Africa. Glob Environ Chang. (2015) 32:187–99. 10.1016/j.gloenvcha.2015.03.005 28149004 PMC5268344

[21] Sassi M Thakare H. Conflict and child malnutrition: a systematic review of the emerging quantitative literature. Curr Nutr Rep. (2022) 11:386. 10.1007/s13668-021-00386-w 35094307

[22] Maxwell D. Famine early warning and information systems in conflict settings: challenges for humanitarian metrics and response. Confl Res Programme. (2019) 2019:30.

[23] Bello GI. The international politics of famine relief operations in Ethiopia: A case study of the 1984-86 famine relief operations (Ph.D thesis). London School of Economics and Political Science (1990).

[24] Haileselassie B Roba KT Weldegebreal F. Undernutrition and its associated factors among pediatric age children attending antiretroviral therapy in Eastern Ethiopia. East African J Heal Biomed Sci. (2019) 3:1–12. Available online at: http://ejol.ethernet.edu.et/index.php/EAJHBS/article/view/1355

[25] Yebyo HG Kendall C Nigusse D Lemma W. Outpatient therapeutic feeding program outcomes and determinants in treatment of severe acute malnutrition in Tigray, Northern Ethiopia: a retrospective cohort study. PLoS ONE. (2013) 8:2–10. 10.1371/journal.pone.0065840 23755286 PMC3675046

[26] Abate BB Tilahun BD Kassie AM Kassaw MW. Treatment outcome of Severe Acute Malnutrition and associated factors among under-five children in outpatient therapeutics unit in Gubalafto Wereda, North Wollo Zone, Ethiopia, 2019. PLoS ONE. (2020) 15:e0238231. 10.1371/journal.pone.0238231 32881883 PMC7470268

[27] Getahun MB Teshome GS Fenta FA Bizuneh AD Mulu GB Kebede MA. Determinants of severe acute malnutrition among HIV-positive children receiving HAART in Public Health Institutions of North Wollo Zone, Northeastern Ethiopia: unmatched case-control study. Pediatr Heal Med Ther. (2020) 11:313–21. 10.2147/PHMT.S267892 32982539 PMC7490065

[28] shitaye Desta K. Survival status and predictors of mortality among children aged 0-59 months with severe acute malnutrition admitted to stabilization center at Sekota Hospital Waghemra Zone. J Nutr Disord Ther. (2015) 5:1–11. 10.4172/2161-0509.1000160

[29] Mengesha MM Deyessa N Tegegne BS Dessie Y. Treatment outcome and factors affecting time to recovery in children with severe acute malnutrition treated at outpatient therapeutic care program. Glob Health Action. (2016) 9:30704. 10.3402/gha.v9.30704 27396484 PMC4939403

[30] CSA. Population Size by Sex, Area and Density by Region, Zone and Wereda Addis Ababa (2013).

[31] USAID. Ethiopia South Omo Zone Conflict Assessment. (2021). p. 9. Available online at: www.democracyinternational.com (accessed December 20, 2022).

[32] Sewale Y Moges NA Zewedie BT Gebeyew AE Giza M. Stunting and its associated factors among human immuno- deficiency virus positive children who receiving anti-retro- viral therapy in Northwest Ethiopia: multicenter study. J AIDS Clin Res. (2021). p. 12.

[33] Jeylan A Mohammed E Girma A. Magnitude of stunting, thinness and associated factors among HIV positive children attending chronic HIV care and support in Adama Hospital Medical College, Adama, Oromia Regional State, Ethiopia. Int J Health Sci Res. (2018) 8:245–56. Available online at: https://www.ijhsr.org/IJHSR_Vol.8_Issue.11_Nov2018/33.pdf

[34] WHO. Global Nutrition Policy Review 2016-2017 Country. Routledge Handbook of Global Public Health. Geneva: WHO (2017). p. 174.

[35] Sutton SS Magagnoli J Hardin JW. Odds of viral suppression by single-tablet regimens, multiple-tablet regimens, and adherence level in HIV/AIDS patients receiving antiretroviral therapy. Pharmacotherapy. (2017) 37:204–13. 10.1002/phar.1889 28028855

[36] Tiruneh CM Walle BG Emiru TD Tibebu NS Abate MW Nigat AB. Under-nutrition and associated factors among children on ART in Southern Ethiopia: a facility-based cross-sectional study. Ital J Pediatr. (2021) 47:1–10. 10.1186/s13052-021-01154-w 34635139 PMC8507210

[37] Ngo NV Pemunta NV Muluh NE Adedze M Basil N Agwale S. Armed conflict, a neglected determinant of childhood vaccination: some children are left behind. Hum Vaccines Immunother. (2020) 16:1454–63. 10.1080/21645515.2019.1688043 31809650 PMC7482736

[38] Martineau T McPake B Theobald S Raven J Ensor T Fustukian S. Leaving no one behind: lessons on rebuilding health systems in conflict- and crisis-affected states. Br Med J Glob Heal. (2017) 2:1–6. 10.1136/bmjgh-2017-000327 29082000 PMC5656126

[39] FAO IFAD UNICEF WFP AW FAO IFAD UNICEF WFP WHO. The State and Food Security and Nutrition in the World 2019. Safeguardin Against Economic Slowdowns and Downturns. Rome: FAO (2019). p. 212.

[40] UN. The 2030 Agenda and the Sustainable Development Goals An opportunity for Latin America and the Caribbean Thank you for your interest in this ECLAC Publication. (2018). p. 1–94. Available online at: https://repositorio.cepal.org/bitstream/handle/11362/40156/25/S1801140_en.pdf (accessed December 20, 2022).

[41] García Cruz LM González Azpeitia G Reyes Súarez D Santana Rodríguez A Loro Ferrer JF Serra-Majem L. Factors associated with stunting among children aged 0 to 59 months from the central region of Mozambique. Nutrients. (2017) 9:1–16. 10.3390/nu9050491 28498315 PMC5452221

[42] Fufa DA. Determinants of stunting in children under five years in dibate district of Ethiopia: a case-control study. Hum Nutr Metab. (2022) 30:200162. 10.1016/j.hnm.2022.200162

[43] Demirchyan A Petrosyan V Sargsyan V Hekimian K. Predictors of stunting among children ages 0 to 59 months in a rural region of Armenia. J Pediatr Gastroenterol Nutr. (2016) 62:150–6. 10.1097/MPG.0000000000000901 26192698

[44] Chowdhury S Chakraborty P. Universal health coverage—There is more to it than meets the eye. J Fam Med Prim Care. (2017) 6:169–70. 10.4103/jfmpc.jfmpc_13_17 PMC5629889 29026777

[45] Mohamed A. Determinants of Malnutrition among under five children in SOS Hospital, Mogadishu. Elixir Int J. (2016). 99:43136–66. Available online at: www.elixirpublishers.com

[46] Berhanu A Garoma S Arero G Mosisa G. Stunting and associated factors among school-age children (5-14 years) in Mulo district, Oromia region, Ethiopia. SAGE Open Med. (2022) 10:20503121221127880. 10.1177/20503121221127880 36212231 PMC9536101

[47] UNICEF. Conceptual Framework on Maternal and Child Nutrition. Nutr Child Dev Sect Program Gr 3 United Nations Plaza New York, NY 10017, USA. New York, NY: UNICEF (2021). p. 2–3.

[48] Ndirangu M Sachs SE Palm C Deckelbaum RJ. HIV affected households in Western Kenya experience greater food insecurity. Food Policy. (2013) 42:11–7. 10.1016/j.foodpol.2013.06.005

[49] Kusum L Wude B. Factors associated with nutritional status of human immunodeficiency virus infected children Hawassa, Ethiopia. Int J Heal Sci Res. (2020) 10:50. Available online at: https://www.ijhsr.org/IJHSR_Vol.10_Issue.5_May2020/10.pdf

[50] Mangili A Murman DH Zampini AM Wanke CA. Nutrition and HIV infection: review of weight loss and wasting in the era of highly active antiretroviral therapy from the nutrition for healthy living cohort. Clin Infect Dis an Off Publ Infect Dis Soc Am. (2006) 42:836–42. 10.1086/500398 16477562

[51] Kristina T Caroline D. Food Systems in Conflict and Peacebuilding Settings: Ways Forward. Stockholm: Sipri (2022).

[52] George J Adelaja A Weatherspoon D. Armed conflicts and food insecurity: evidence from Boko Haram's Attacks. Am J Agric Econ. (2020) 102:114–31. 10.1093/ajae/aaz039

[53] Molla A Degu T Asmare WN Bekalu A Girma A Derjie R. Dietary diversity and associated factors among HIV positive children age 6 months to 14 years in southwest Ethiopia: institution based cross-sectional study. Heliyon 24.

[54] Mekonen J Addisu S Mekonnen H. Prevalence and associated factors of chronic undernutrition among under five children in Adama town, Central Ethiopia: a cross-sectional study design. BMC Res Notes. (2019) 12:1–6. 10.1186/s13104-019-4552-1 31429806 PMC6702749

[55] Teshome B Kogi-Makau W Getahun Z Taye G. Magnitude and determinants of stunting in children under five years of age in food surplus region of Ethiopia: the case of West Gojam Zone. Ethiop J Heal Dev. (2010) 23:53223. 10.4314/ejhd.v23i2.53223

[56] Mashal T Takano T Nakamura K Kizuki M Hemat S Watanabe M. Factors associated with the health and nutritional status of children under 5 years of age in Afghanistan: family behaviour related to women and past experience of war-related hardships. BMC Public Health. (2008) 8:301. 10.1186/1471-2458-8-301 18759988 PMC2551613

[57] Miller TL Orav EJ Martin SR Cooper ER McIntosh K Winter HS. Malnutrition and carbohydrate malabsorption in children with vertically transmitted human immunodeficiency virus I infection. Gastroenterology. (1991) 100:1296–302. 10.1016/0016-5085(91)70016-Q 2013374

[58] Yolken RH Hart W Oung I Shiff C Greenson J Perman JA. Gastrointestinal dysfunction and disaccharide intolerance in children infected with human immunodeficiency virus. J Pediatr. (1991) 118:359–63. 10.1016/S0022-3476(05)82147-X 1999774

[59] Zuin G Fontana M Nicoli S Scapellato L Tamburini G Gaboardi F. Persistence of protein loss in acute diarrhoea. A follow-up study by faecal alpha-1-antitrypsin measurement. Acta Paediatr Scand. (1991) 80:9613. 10.1111/j.1651-2227.1991.tb11761.x 1755305

[60] Friend CJ Heard CRC Platt BS Stewart RJC Turner MR. The effect of dietary protein deficiency on transport of vitamin A in the blood and its storage in the liver. Br J Nutr. (1961) 15:231–40. 10.1079/BJN19610028 13702061

[61] Bender DA. Effects of a dietary excess of leucine on the metabolism of tryptophan in the rat: a mechanism for the pellagragenic action of leucine. Br J Nutr. (1983) 50:25–32. 10.1079/BJN19830068 6882730

[62] Roels OA. Vitamin a and protein metabolism. N Y State J Med. (1964) 64:288–94. 14103682

[63] Hambidge KM. Zinc and diarrhea. Acta Paediatr. (1992) 81:82–6. 10.1111/j.1651-2227.1992.tb12377.x 1421947

[64] Reddy V Raghuramulu N Arunjyoti Shivaprakash M Underwood B. Absorption of vitamin A by children with diarrhoea during treatment with oral rehydration salt solution. Bull World Health Organ. (1986) 64:721–4. 3492305 PMC2490959

[65] Sivakumar B Reddy V. Absorption of labelled vitamin A in children during infection. Br J Nutr. (1971) 27:299–304. 10.1079/BJN19720094 5015249

[66] Sommer A. Xerophthalmia and vitamin A status. Prog Retin Eye Res. (1998) 17:9–31. 10.1016/S1350-9462(97)00001-3 9537797

[67] Murray CJL King G Lopez AD Tomijima N Krug EG. Armed conflict as a public health problem. Br Med J. (2002) 324:346–9. 10.1136/bmj.324.7333.346 11834565 PMC1122272

[68] Gayer M Legros D Formenty P. Connolly MA. Conflict and emerging infectious diseases. Emerg Infect Dis. (2007) 13:1625–31. 10.3201/eid1311.061093 18217543 PMC3375795

[69] Action T. Meganav—EN (2019).

